# Wire Arc Additive Manufacturing (WAAM) of Aluminum Alloy AlMg5Mn with Energy-Reduced Gas Metal Arc Welding (GMAW)

**DOI:** 10.3390/ma13122671

**Published:** 2020-06-12

**Authors:** Maximilian Gierth, Philipp Henckell, Yarop Ali, Jonas Scholl, Jean Pierre Bergmann

**Affiliations:** Production Technology Group, Technische Universität Ilmenau, D-98693 Ilmenau, Germany; philipp.henckell@tu-ilmenau.de (P.H.); yarop.ali@tu-ilmenau.de (Y.A.); jonas.scholl@tu-ilmenau.de (J.S.); jeanpierre.bergmann@tu-ilmenau.de (J.P.B.)

**Keywords:** additive manufacturing, aerospace, aluminum, automotive, GMAW, grain size, homogenous properties, microstructure, temperature, wire arc additive manufacturing

## Abstract

Large-scale aluminum parts are used in aerospace and automotive industries, due to excellent strength, light weight, and the good corrosion resistance of the material. Additive manufacturing processes enable both cost and time savings in the context of component manufacturing. Thereby, wire arc additive manufacturing (WAAM) is particularly suitable for the production of large volume parts due to deposition rates in the range of kilograms per hour. Challenges during the manufacturing process of aluminum alloys, such as porosity or poor mechanical properties, can be overcome by using arc technologies with adaptable energy input. In this study, WAAM of AlMg5Mn alloy was systematically investigated by using the gas metal arc welding (GMAW) process. Herein, correlations between the energy input and the resulting temperature–time-regimes show the effect on resulting microstructure, weld seam irregularities and the mechanical properties of additively manufactured aluminum parts. Therefore, multilayer walls were built layer wise using the cold metal transfer (CMT) process including conventional CMT, CMT advanced and CMT pulse advanced arc modes. These processing strategies were analyzed by means of energy input, whereby the geometrical features of the layers could be controlled as well as the porosity to area portion to below 1% in the WAAM parts. Furthermore, the investigations show the that mechanical properties like tensile strength and material hardness can be adapted throughout the energy input per unit length significantly.

## 1. Introduction

Aluminum and its alloys can be used in a wide variety of applications due to their combination of favorable properties. They are characterized by low density, favorable strength and deformation properties as well as high thermal and electrical conductivity. In addition, aluminum alloys exhibit high corrosion resistance, good weathering and chemical resistance due to the development of a passivating oxide layer [1,2].

Based on the special requirements of the aerospace, automotive and tool-making industries, the production of complex metal components including aluminum, titanium and nickel alloys has become a major focus in recent years. Due to its low cost and material properties, aluminum in particular is of great relevance for the production of large-volume lightweight components. Subtractive manufacturing methods such as milling or turning reach their technical process limits in terms of maximum available space or fabrication of undercuts in complex components. This also results in a high chip volume, which reduces the efficiency of the processes.

In recent years, additive manufacturing processes have gained increasing importance in industrial applications due to specific advantages such as material utilization, freedom of design and timesaving [3,4]. A suitable approach for the additive manufacturing of large volume components is the usage of arc-based manufacturing processes. In particular, wire arc additive manufacturing (WAAM) with gas metal arc welding (GMAW) is of high interest due to high deposition rates, material efficiency > 90% and the almost unlimited space available [5]. In addition, WAAM offers the possibility to create complex geometries, which allows the production of novel, lightweight structures [6]. Although, the manufactured parts exhibit surface roughness, which requires post processing with subtractive methods [7,8].

However, WAAM of aluminum materials is limited by defects such as porosity and solidification cracks [9,10]. Porosity is the main problem and can severely limit the mechanical properties of the components, such as component strength or ductility [11,12]. In contrast to single-pass welding, the deposited metal is built layer-wise during additive manufacturing. Accordingly, the heat input acts as a low-temperature heat treatment for the previously deposited layer, which influences the growth of pores, especially in age-hardenable aluminum alloys [12]. However, the porosity, the microstructure, the mechanical properties and the final contour of aluminum parts depend to a large extent on the choice of the arc-welding process, the process parameters, such as the welding speed, and the resulting heat input [9,11,13,14,15]. Porosity is also affected by other factors such as wire quality and alloy composition [16,17], as well as interpass temperature [18,19], and depends on the microstructure being formed [11,20].

An established method for WAAM with GMAW is the cold metal transfer (CMT) process from Fronius and its variations in arc mode. Herein, polarity reversal of the welding current (CMT advanced (CMT-ADV)) or the simultaneous pulse superimposition of the base current during positive polarity of the wire electrode (CMT-pulse advanced (CMT-PADV)) [21] can effectively reduce the heat input [22]. The influence of different CMT arc modes and process parameters on porosity is of high interest in scientific publications [11,14,20,23]. Fang et al. found that in welding with S Al 2319, the pore size and the spatial distribution of the pores in the generated geometry are of relevance, in addition to the proportion of pores in the total area. The area percentage varies considerably among the processes and is lowest with 0.98% for the CMT-PADV arc [14]. Cong et al. conducted investigations with the same material and came to the same results regarding the lowest porosity in the CMT-PADV process [11]. As a result, the porosity can be reduced by lowering the energy input due to a reduction of the peak temperature and the shortened residence time at high temperatures [11,14]. Simultaneously, it was found that the porosity in block structures is lower than in thin-walled structures. This is due to a higher heat dissipation through a larger cross-sectional area and results in a smaller number of pores and a finer microstructure [13]. Further investigations on the influence of the CMT process in WAAM were carried out for aluminum alloys of the groups 2xxx [24], 6xxx [25] and 7xxx [26].

The group of aluminum–magnesium alloys are characterized by medium to high strength, corrosion resistance and good fatigue properties. They are highly cold-workable and can be easily welded if the magnesium content exceeds 3%. Al–Mg alloys are mainly rolled products. In addition, they are used for the production of bars, tubes and wires, and are processed into drop forgings and open-die forgings. Therefore, aluminum–magnesium alloys are part of the most important construction alloys [27]. Derekar et al. compared the influence of the impulse arc process and the standard CMT process on hydrogen solubility and thus on the formation and distribution of pores in the aluminum alloy S Al 5183. It was found that lower porosities could be achieved with the CMT process [28]. Li et al. investigated the influence of pure argon and pure nitrogen as a shielding gas on the resulting geometrical and technical properties of the alloy S Al 5356 by using a short arc WAAM process [29].

Another representative of this group with industrial relevance is the alloy AlMg5Mn (S Al 5556). This alloy is used in automotive, wagon and ship building due to its high strength. Example applications are stiffeners and seat cushion frames in automotive engineering [30]. To date, no studies have been published on this material in the context of WAAM with GMAW. Accordingly, there is no information on the correlation of the CMT arc mode used and the process influencing variables with the resulting microstructure and porosity. The same applies to the effects on the achievable mechanical properties.

## 2. Scope of the Investigations

The aim of this study was the systematic investigation of the aluminum alloy AlMg5Mn in wire- and arc-based additive manufacturing. For this purpose, gas-shielded metal arc welding was applied using the energy reduced and controlled short-arc technology. The focus is on the analysis of the influence of the considered arc modes CMT, CMT advanced (CMT-ADV) and CMT-pulse Advanced (CMT-PADV) on the energy input per unit length, the temperature–time curve and the resulting final contour, microstructure and mechanical-technological properties. Thus, the selection of the appropriate arc mode and the specific adjustment of the welding parameters should enable the additive production of flawless primary structures. In order to achieve the goal, the process parameters, such as wire feed or welding speed, were first varied in preliminary tests and small volume structures were welded. Subsequently, the porosity of the samples was analyzed and the final contour was visually assessed. Subsequently, the CMT process, which was used to achieve the lowest porosity, was used to investigate buildup strategies for further reducing the porosity and increasing the final contour accuracy and avoiding bonding defects. Subsequently, a large-volume wall structure was built up using the CMT processes in the main tests and examined and compared with regard to the achieved final contour, the microstructure and the mechanical-technical properties. Thus, a recommendation for the most suitable arc mode for the additive manufacturing of AlMg5Mn using WAAM can be derived.

## 3. Materials and Methods

**Figure 1:**
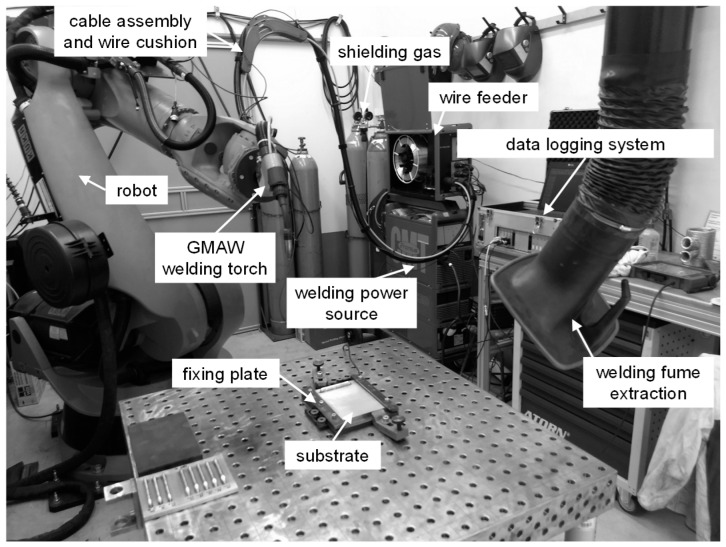
Experimental setup for the experimental trials using gas metal arc welding (GMAW).

The experimental trials were carried out with a robot-supported experimental setup as shown in Figure 1. The basic components for the welding process were a CMT Advanced 4000 R welding power source (Fronius Deutschland GmbH, Neuhof-Dorfborn, Germany), a VR 7000-CMT wire feeder with 4-roller drive (Fronius Deutschland GmbH, Neuhof-Dorfborn, Germany), a Robacta Drive CMT push-pull robot welding torch (Fronius Deutschland GmbH, Neuhof-Dorfborn, Germany) and a hose package with wire buffer (Fronius Deutschland GmbH, Neuhof-Dorfborn, Germany). As the handling system, a KUKA KR 150-2 robot (KUKA Aktiengesellschaft, Augsburg, Germany) was used. For the additive buildup process, the substrate was fixed on a welding table. The welding power source was a digitally regulated GMAW power source, which enabled welding with the three energy-reduced arc modes CMT, CMT-ADV and CMT-PADV, which were used in this investigation. The use of the KUKA six-axis robot provided an accurate and repeatable torch movement with a positional repeatability of 0.06 mm.

The welding current, the arc voltage, the measurement data for determining the wire-feed speed as well as the temperature on the surface of the structure at the defined measuring points during the buildup process were acquired by a Dewetron DEWE-PCI 16 measuring system (Dewetron GmbH, Grambach, Austria) with measuring cards, recorded and analyzed with the integrated data acquisition software DEWESoft 7.1.1 (DEWESoft Deutschland GmbH, Unterensingen, Germany). An EWM MWSTROM measuring box was integrated into the welding circuit to measure the current intensity. Using the measured welding currents and arc voltages, the line energy can be calculated based on Equation (1), where *E_s_* is the energy input per unit length, *U* is the arc voltage, *I* is the welding current and *v_s_* is the welding speed. Since there are no constant current and voltage values for the arc regime used and the average values falsify the result, the time-dependent Equation (2) is used. Irrespective of the arc mode, the same time interval is integrated for the calculation of the energy input per unit length:

(1) $$E_{S}\left[\frac{kJ}{cm}\right]=\frac{U\cdot I}{v_{S}}\left[\frac{VA}{\frac{cm}{s}}\right]$$

(2) $$E_{S}=\frac{\frac{1}{\left(t_{n}-t_{0}\right)}\sum_{i=0}^{n}\int_{t_{i}}^{t_{i}+1}u_{i}\cdot i_{i}\;dt}{v_{S}}$$

**Figure 2:**
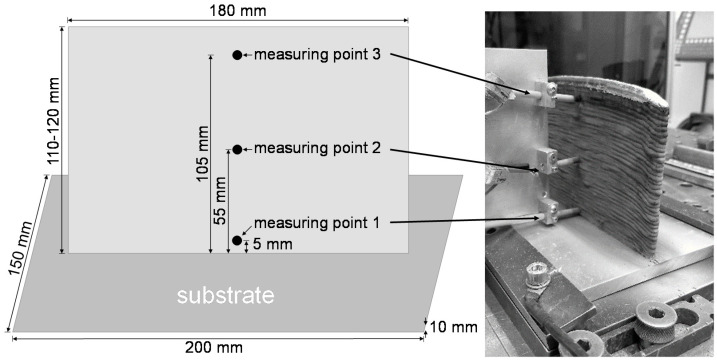
Representation of the placement of the temperature measuring points and dimensions of the substrate and the built wall structures.

For the analysis of the temperature–time curve during the additive buildup process, three temperature measuring points in the wall structures were distributed over the progress of the process at defined positions. As shown in Figure 2, the measuring points are located in a horizontal direction in the middle of the structure. In the buildup direction, the first measuring point was placed directly at the height of the first weld bead (approx. 5 mm) in order to measure the cooling rate in close proximity to the substrate, to analyze when the substrate reached thermal equilibrium and what temperature was reached in this state. The second measuring point was placed at a distance of approx. 55 mm from the substrate in the buildup direction. Here, the temperature–time curve in the continuous buildup process was monitored and recorded, whereby changes in the cooling rate were suspected. At the same time, the establishment of a thermal equilibrium could be observed. The third measuring point was located in the upper area of the wall structure at a distance of approx. 105 mm from the substrate surface and allows an investigation of the temperature–time curve at this late stage of the additive buildup process (little reheating).

Type C thermocouples with a diameter of 1 mm were used to record the temperature curve. They were placed on the substrate or welding bead at the corresponding measuring points and overwelded. In addition, the process was monitored with an ImageIR 8300 thermal imaging camera (InfraTec GmbH, Dresden, Germany) and the cooling behavior in the areas of the measuring points was recorded and analyzed using the IRBIS 3.1 plus analysis software (InfraTec GmbH, Dresden, Germany).

The interpass temperature was measured with a type K sheath thermocouple with a diameter of 1 mm in combination with a 4-channel data logger MCR-4TC (T&D Corporation, Matsumoto, Japan) in contact in the middle of the last welded bead.

In this research, a welding wire of the type S Al 5556 with a diameter of 1 mm was used as a consumable electrode for the GMAW process. The aluminum alloy EN AW-5754A H111 in the form of a plate with the dimensions 200 × 150 × 10 mm^3^ (see Figure 2) was used as a substrate. The chemical composition of the welding wire and the substrate material are listed in Table 1.

Both argon 4.6 with a flow rate of 20 L/min and a mixture of argon and helium in a ratio of 70/30 with an adjusted flow rate of 24 L/min were used as a shielding gas. Argon was used because of its good shielding effect due to its high density. As a result of the lower density of helium, the flow rate had to be adjusted when using the gas mixture. Due to the amount of helium, the energy input into the molten bath can be increased, which influenced the formation of the beads.

**Figure 3:**
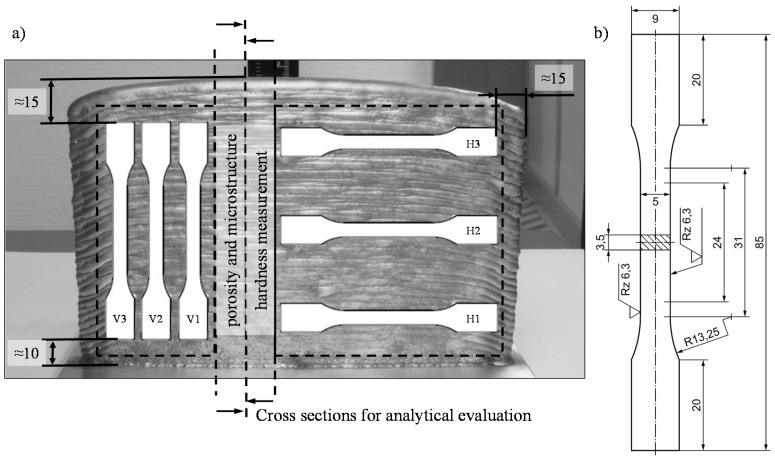
(**a**) Schematic representation of the arrangement of the tensile specimens vertically (V) and horizontally (H) to the buildup direction, as well as the wall segments to evaluate hardness, porosity and the final contour and (**b**) dimension of the tensile specimen of form E according to DIN 50125:2016-12.

The investigated wall structures had a minimum height of approx. 110 mm and a length of 180 mm (see Figure 2), and were generated with an alternating buildup strategy. The separation from the substrate was carried out with a band saw. Subsequently, wall segments were cut off for the analysis of the microstructure, hardness and final contour. For the determination of the tensile strength, the remaining wall segments were milled over so that a planar surface with a thickness of 3.5 mm was created and tensile specimens of form E according to DIN 50125:2016-12 (see Figure 3b) were cut out by wire-electro discharge machining (EDM). The tensile specimens were removed in the buildup direction (vertical) and in the welding direction (horizontal). The arrangement of the different specimens and the minimum distance of the edge of the structure are shown in Figure 3a.

The samples for the analysis of the geometric properties and microstructure were grinded and polished. To investigate the microstructure, an electrolytic etch polishing and an etching according to Barker were additionally carried out. The sample structures were then examined under polarized light. The grain size determination was carried out according to the standardization for steels by DIN EN ISO 643 using the line section method. The result was the determination of the average linear grain size.

**Figure 4:**
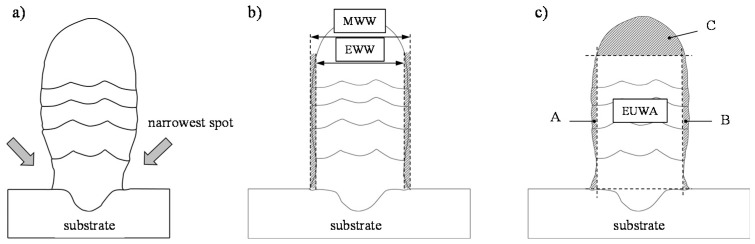
Schematic representation of (**a**) the characteristic development of the bead width with increasing structure height, (**b**) the maximum wall width MWW and effective wall width EWW and (**c**) the effective usable wall area EUWA.

In order to assess the final contour, the material utilization (MU) and the surface irregularity (SI) of the lateral surfaces of the cross-sections were measured. As the number of layers increases, the width of the molten bath in the WAAM changes due to the heat accumulation and the change in the heat transfer (see Figure 4a). To calculate the surface irregularity of the side surfaces, the effective wall width (EWW) and the maximum wall width (MWW) were measured (see Equation (3) and Figure 4b). EWW is the maximum effectively usable width of the wall. The surface irregularity *SI* of the side surfaces was calculated by the quotient (MWW-EWW)/2. On both sides, the span between the narrowest point and the highest vertex in the vertical profile of the side surface in the cross-section of the sample was added and divided by two. Figure 4c schematically shows the effective usable wall area (EUWA) of the generated wall. Areas A, B and C must be removed after additive manufacturing for most applications. However, area C is not considered in this paper when calculating the material utilization, as it always occurs regardless of the number of layers and must be removed subtractively. Consequently, this area has a greater influence with a small number of welded layers than with a large number of layers. The quotient EUWA/(EUWA + A + B) indicates the maximum material utilization with the full utilization of the EWW, considering that area C of the wall was not measured (see Equation (4)):

(3) $$SI=\frac{\left(MWW-EWW\right)}{2}$$

(4) $$MU=\frac{EUWA}{\left(EUWA+A+B\right)}$$

**Figure 5:**
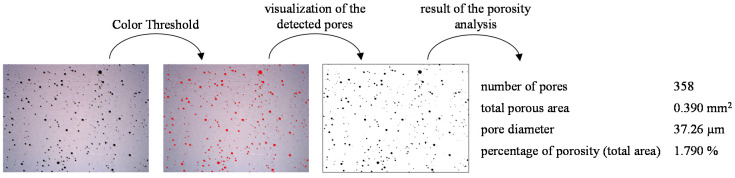
Exemplary representation of the process of porosity analysis.

In the present study, the porosity measurement was carried out by means of microsectioning and sectional testing on the metallographically prepared sample. The porosity was determined using the open source image-processing program ImageJ (version 1.52e) on the basis of the panoramic images of the prepared specimens, which were taken at a magnification of 25:1 under the Axio Scope.A1 (Carl Zeiss Microscopy GmbH, Jena, Germany) incident light microscope. The procedure for evaluating the porosity is shown as an example in Figure 5.

For the porosity analysis, the scale was initially set according to the magnification of the microscope. Then, an upper brightness threshold was set with respect to the color thresholds using the brightness slider so that the color values were filtered according to the brightness of the color values of the pores and displayed in the selected color red. The selection of the contour of the wall was made including the weld penetration, but without the rest of the substrate. The analysis tool was used to measure the area of the selected contour. Then, the number of pores, the total pore area, the average pore size and the percentage of porosity in the total area were determined. Due to the resolution of the optical microscope, only pores with a diameter greater than 10 µm were measured.

The tensile test was performed with a universal testing machine inspekt retrofit 1455 (Hegewald & Peschke Meß- und Prüftechnik GmbH, Nossen, Germany) according to DIN EN ISO 6892-1 at a testing speed of *v_c_* = 10 mm/min and an estimated strain rate of *ė*_Lc_ = 0.0054 s^−1^. The strain rate was estimated based on the parallel length *L_c_* = 31 mm and the constant testing speed due to the inhomogeneous flow of the material during the tensile tests. The hardness was determined on the cross-sectional areas along the wall height by using a hardness test according to Vickers (DIN EN ISO 6507-1) in the small force range with a force of 9.807 N (HV1). The imprints have a distance of 1 mm from each other. The measurement was done with an automatic hardness testing machine DuraScan 70 (EMCO-TEST Prüfmaschinen GmbH, Kuchl, Österreich).

## 4. Results and Discussion

### 4.1. Preliminary Analysis of the CMT Process with Different Arc Modes

In the preliminary investigations, the influence of the arc modes, the welding speed and consequently the energy input per unit length on the porosity, wall geometry, final contour and the connection to the substrate were investigated by using the CMT, CMT-ADV and CMT-PADV arc mode. For this purpose, four five-layer wall structures were additively manufactured with each arc mode. The wire feed rate was kept approximately constant. A variation of the energy per unit length was achieved by changing the welding speed between 0.3 and 0.6 m/min. With regard to the ratio of positively/negatively poled arc cycles in the CMT-ADV or CMT-PADV process, the starting point was defined as the ratio of seven positive cycles followed by seven negative cycles as stored in the welding characteristic curve of the power source.

**Figure 6:**
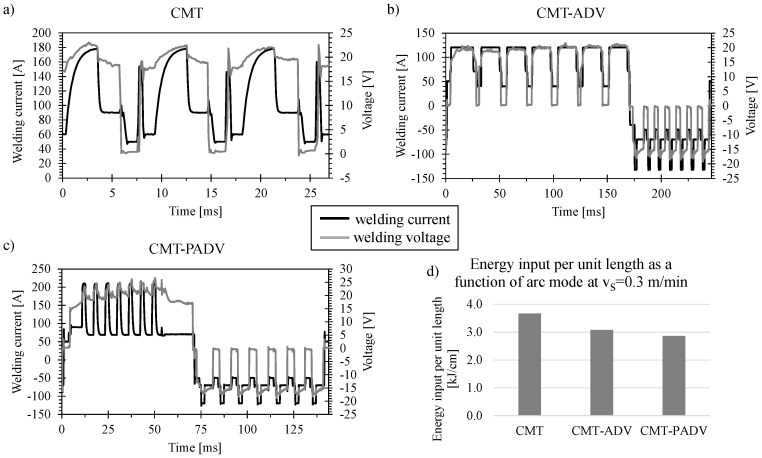
Current and voltage characteristics of (**a**) Cold Metal Transfer (CMT), (**b**) CMT advanced (CMT-ADV) and (**c**) CMT pulse advanced (CMT-PADV) and (**d**) the energy input per unit length as a function of the arc mode at a welding speed of *v_S_* = 0.3 m/min.

Figure 6 shows the current and voltage characteristics for (a) the CMT, (b) the CMT-ADV and (c) the CMT-PADV arc mode. The curves were measured at a welding speed of 0.3 m/min and the determined wire-feed speeds of 9.45 m/min for CMT, 9.64 m/min for CMT-ADV and 9.26 m/min for CMT-PADV. The slight deviations in the measured wire feed speed can be explained by the different arc modes and the appropriate integrated control of the welding power source. Figure 6b shows the cyclical polarity change of the welding current during the short circuit phase, which is a characteristic feature of the CMT-ADV process. The pre-set ratio of seven positive cycles followed by seven negative cycles can also be seen. The material transfer during both positive and negative polarity occurs in the short circuit phase. Negative polarity, in contrast to positive polarity, increases the arcing point, which increases the deposition rate despite the lower heat input. During the positive cycle, more heat was introduced into the base material (for bonding) and cleaned the workpiece surface of the aluminum oxide layer. The CMT-PADV process in Figure 6c illustrates the combination of negative-polarity CMT cycles with cyclic wire movement and positive-polarity pulse cycles with continuous wire feed. Here, seven negatively poled CMT cycles follow seven positively poled pulse cycles. In the pulse cycles, the material transfer is short-circuit-free and allows a higher heat input into the base material. Higher deposition rates can be achieved than in the CMT-ADV process [21].

Figure 6d shows the resulting energy input per unit length at a welding speed of 0.3 m/min. Using the CMT arc mode, an energy input per unit length of 3.67 kJ/cm was obtained. Due to the changing polarity in the CMT-ADV arc mode, the energy input was reduced to 3.08 kJ/cm. The lowest energy input per unit length of 2.86 kJ/cm could be measured in the CMT-PADV arc mode and results from the positively poled pulsed current cycles in combination with the alternating polarity. Since the heat input correlates with the energy input per unit length, it can be assumed that the heat input into the welding process in CMT-ADV or CMT-PADV arc mode can be reduced.

**Figure 7:**
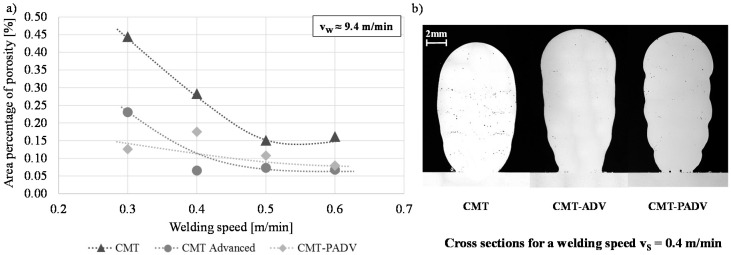
(**a**) Correlation of the porosity and the welding speed for the arc modes CMT, CMT-ADV and CMT-PADV with a wire-feed speed of approx. *v_w_* = 9.4 m/min and (**b**) the optically observed porosity in the cross-sections of the five-layer wall structures for the different arc modes at a welding speed of *v_S_* = 0.4 m/min.

Figure 7a shows the porosity in the cross-sections of the additive manufactured walls made of AlMg5Mn that were investigated as a function of the welding speed and the used arc mode. During the trial series the wire-feed speed was kept constant at approx. *v_w_* = 9.4 m/min. The measured area percentage of porosity was the highest at all four welding speeds in the standard CMT process. At *v_S_* = 0.4 m/min, the observed porosity was more than four times as high compared to the CMT-ADV process at the same speed. Using the CMT-ADV arc mode, the lowest porosity was observed, apart from the welding speed of *v_S_* = 0.3 m/min. While the porosity was already at a very low level when using the CMT-ADV mode at a welding speed of 0.4 m/min, and remained almost unchanged at around 0.07% at higher speeds, the other two arc modes show a decrease in porosity as the welding speed increases. The thus reduced energy input per unit length and the associated reduction in heat input have a corresponding porosity reducing effect.

In addition, in the CMT-ADV and CMT-PADV processes, a significant oxide cleaning effect was achieved at the wire electrode end during the cycles with negative electrode polarity. This reduces the hydrogen content entering the melt pool. The stirring effect caused by the change in polarity and the resulting turbulence in the melt pool leads to grain refinement and has a beneficial effect on the escape of pores [15]. The high percentage of porosity in the CMT-PADV process at a welding speed of 0.4 m/min is caused by the two large pores that have formed between the first layer and the substrate. These can be seen in the comparison of the structures at a welding speed of 0.4 m/min in Figure 7b. As the entire structure was examined for porosity, these are part of the analysis. A possible reason for the pores is a polluted substrate surface.

It could be detected that the pores were mainly distributed at the boundaries between the individual layers. In addition, an insufficient weld penetration and/or lack of bonding between the substrate and the first layer of the walls can be observed. The bonding to the substrate and thus the relatively small bead width of the first layer is most significant in the CMT-PADV process with the lowest heat input.

### 4.2. Preliminary Analysis of the CMT-ADV to Improve Final Contour and Porosity

**Figure 8:**
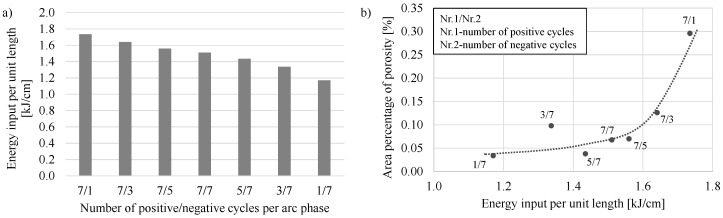
Correlation between (**a**) the energy input per unit length and (**b**) the porosity and the energy input per unit length (assigned with the number of positive and negative cycles per arc phase) at a welding speed of 0.6 m/min.

In further experiments, the influence of the varying ratios of positively and negatively poled current cycles within an arc phase on energy input per unit length, surface irregularity, material utilization and porosity were investigated using the CMT-ADV arc mode. Starting from a balanced ratio of seven positive and seven negative cycles, the number of positive and negative cycles was systematically reduced by two to a minimum of one while the number of other polarity cycles was kept at seven. As can be seen in Figure 8a, a gradual reduction of the positive cycles in relation to the negative cycles led to a successive decrease in the energy input per unit length at a welding speed of 0.6 m/min. With a constellation of 7/1 cycles, an energy input per unit length of 1.73 kJ/cm can be measured. In contrast, one positive cycle and seven negative cycles result in a line energy of 1.17 kJ/cm. This is a reduction of about 33%.

The correlation between the number of positive and negative cycles per arc phase and the determined area percentage of porosity at a welding speed of 0.6 m/min is shown in Figure 8b. Apart from the constellation 3/7, the area proportion of porosity decreased with the decrease in the quotient of the number of positive cycles to the number of negative cycles and consequently with a reduction of the energy input. The decrease in the area proportion of porosity is due to a decrease in the pore density and a decrease in the average pore size when the number of negative cycles increases (from 7/1 to 7/5). When the number of positive cycles is gradually reduced (from 7/7 to ultimately 1/7), the decrease in porosity, apart from the outlier at 3/7 cycles, is exclusively due to a decrease in pore size. The decrease in porosity with the gradual increase in the negative cycles in relation to the positive ones, from a constellation with 7/1 cycles to 1/7 cycles, can also be attributed to the reduction of the energy input and thus to a reduced heat input.

**Figure 9:**
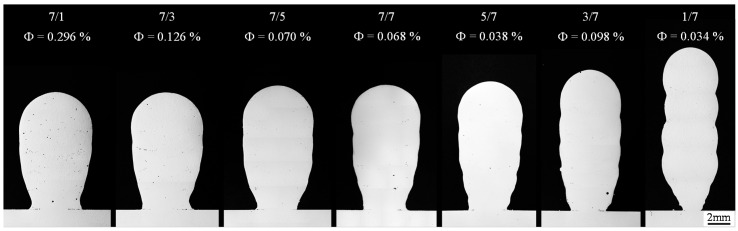
Optically observed porosity (*Φ*) and the final contour of the five-layer walls as a function of the number of positive and negative cycles per arc phase in the CMT-ADV arc mode (*v_S_* = 0.6 m/min).

Figure 9 shows the cross-sections of the samples produced with different numbers of cycles per arc phase and a welding speed of 0.6 m/min. For each constellation of cycles analyzed, the porosity *Φ* is given as a percentage of the measured pore area on the examined cross-section. It can be seen that the gradual change in the cycle constellation from seven positive cycles and one negative cycle to one positive cycle and seven negative cycles has led to a continuous reduction in the wall width and to an increase in wall height. In addition, a trend towards a decrease in the weld penetration area and depth was observed. These phenomena are due to the reduction in energy input per unit length and thus the reduced heat input. A colder melt pool is less flowable and solidifies faster. This results in welding beads with a comparatively large height to width ratio. At the same time, the penetration is reduced due to the faster heat dissipation in the substrate. Accordingly, the weld beads are bonded to the substrate on a small area. A narrow first layer reduces the heat dissipation to the substrate by reducing the heat conduction surface. Furthermore, too little penetration due to component distortion can lead to the wall peeling off the substrate plate.

The highest surface irregularity at 1.7 mm was measured for the constellation of one positive cycle and seven negative cycles. In addition, with this number of positive and negative current phases per arc phase, the material utilization was lowest at just under 37%, which was due to a very narrow first layer. The gradual change in the cycle constellation towards one positive cycle and seven negative cycles led to a gradual increase in the surface irregularity and a decrease in the material utilization. Accordingly, the surface irregularity of the side surfaces was lowest within the constellation of one negative cycle and seven positive cycles. In addition, the material utilization was highest in this cycle constellation at around 75%. A reduction of the positive cycles in relation to the negative cycles also led to a decrease in both the penetration depth and the penetration area.

**Figure 10:**
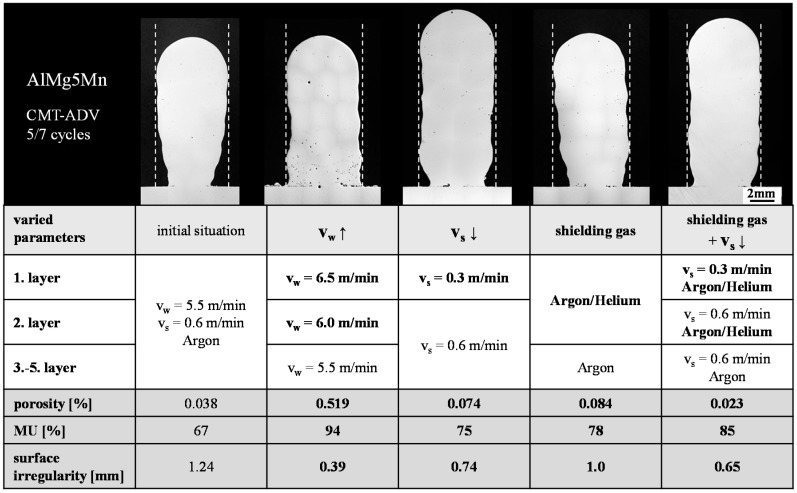
Comparison of the different strategies to increase the final contour accuracy (represented by material utilization (MU) and surface irregularity) of the CMT-ADV arc mode with a cycle constellation of 5/7.

To increase the final contour, various strategies were investigated, which generally involved an increasing energy input as well as the melting rate for the first layers. This was realized by decreasing the welding speed, increasing the wire-feed rate and/or using the argon–helium mixture. The final contour accuracy was evaluated based on the surface irregularity as well as the material utilization (MU) in the cross-section of the samples (see Section 4 Figure 4). The structure manufactured with the CMT-ADV arc mode with five positive and seven negative cycles per arc phase, the shielding gas argon, the welding speed *v_S_* = 0.6 m/min and the wire-feed speed *v_w_* = 5.5 m/min was used as the reference sample. The cycle constellation of five positive cycles followed by seven negatively poled cycles was selected because the lowest porosity and the highest possible material utilization was achieved in the previously conducted investigations under the variation of the cycle configuration (see Figure 9). The constellation of one positive and seven negative poled arc cycles was not used due to the low material utilization. Starting from the parameter of the reference specimen, a reduction in the welding speed when applying the first layer, combined with the use of 70% argon and 30% helium within the first two layers as the only strategy, led to both an improvement in the final contour in combination with a reduction in porosity. The results are summarized in Figure 10.

An increase in the wire-feed rate within the first two layers led to the lowest waviness and the highest material utilization of 94%. However, this resulted in a significant increase in porosity and a decrease in penetration. In addition, a significantly increased spatter formation was observed. The reduction of the welding speed and the use of the argon–helium mixture also led to an improvement of the final contour compared to the initial situation. However, these two strategies also resulted in an increase in porosity. Furthermore, a visual inspection of the wall geometries showed, that all four strategies are suitable for additive manufacturing. The wall width and height were uniform over the length of all four specimens.

The strategy, which combines the lowest porosity and the highest material utilization, can be shown throughout reduced welding speed in the first layer and the adapted shielding gas mixture of 70% argon and 30% helium within the first two layers. This shows a contradiction to the conclusions reached so far. Up to this point, an increase in the energy input resulted in an increase in porosity. The opposite can be observed in the shown build-up strategy. The contradiction may be described throughout the combination of the resulting effects. By reducing the welding speed, a comparatively higher amount of material is molten. By using the shielding gas mixture, the helium content increases the heat of the melt and enhances degasification. As a result, a lack of fusion between the substrate and the first layer can be prevented and the porosity reduced (see Figure 10). Accordingly, this buildup strategy for the first layers is to be favored for the additive manufacturing of AlMg5Mn using the CMT-ADV arc mode.

### 4.3. Analysis of Geometrical Properties of Multi-Layered Structures

Based on the conclusions of the preliminary investigations, large volume wall structures were generated with the CMT, CMT-ADV and CMT-PADV arc modes. The following Sections describe the resulting properties in terms of geometry (see Section 4.3), temperature–time curve (see Section 4.4), porosity (see Section 4.5), microstructure (see Section 4.6) and the selected mechanical properties (see Section 4.7).

The walls have a length of 180 mm and a height of at least 110 mm. The contact tube to work distance was set to 15 mm, periodically checked during the construction process and adjusted if necessary. An alternating buildup strategy was used for the process, i.e., the welding direction was reversed after each generated layer. The interpass temperature was set at 50 °C. To compare the structures with each other, the same welding parameters were used as far as possible. The wire-feed rate was adjusted so that a value of approx. 9.5 m/min could be measured. A welding speed of 0.6 m/min was used. This has been reduced to 0.3 m/min due to the results obtained with regard to the improvement of the final contour and the connection in the first two layers (see Figure 9). For reasons of better comparability, a cycle constellation of 7/7 was used for the CMT-ADV and CMT-PADV arc modes. The reason for the selection of the standard cycle splitting (seven positive and seven negative cycles) is due to the fact that, with regard to the CMT-PADV arc mode, no more in-depth investigations were carried out during the preliminary analysis to improve the final contour and porosity, as they were not part of the project. Argon 4.6 was used as a shielding gas.

**Figure 11:**
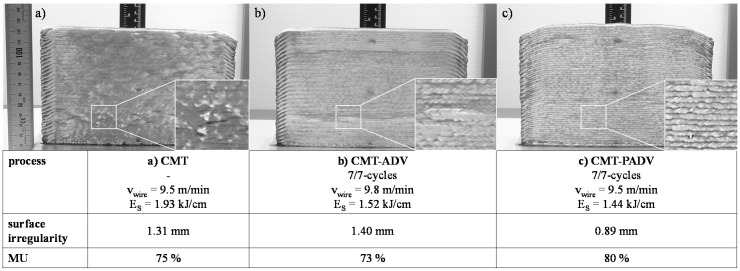
Side view of the multilayer walls made of AlMg5Mn, which were additively manufactured (**a**) by CMT with 62 layers, (**b**) by CMT-ADV with 56 layers and (**c**) by CMT-PADV with 56 layers and the information to the surface irregularity and material utilization (MU).

The multilayer walls built up by means of (a) CMT, (b) CMT-ADV and (c) CMT-PADV are shown in Figure 11. The wall structure built up with the CMT process has a decreasing height in the welding direction. The difference in height between the beginning of the structure and the end is approx. 5 mm. On the one hand, the deviations are due to a significantly higher cooling rate at the beginning of the deposition of a layer, which leads to an increased layer height. On the other hand, low heat dissipation at the end of the layer and the arc pressure lead to a decrease in the layer height. This effect could not be completely compensated by the alternating buildup strategy.

The wall structure produced with the CMT-PADV mode shows a wavy height profile and a comparatively strong drop in height directly at the beginning or end of the structure. A possible reason for the wavy shape is the different amount of melted material between the positively poled and pulsed current phase and the negative poled CMT current phase. Accordingly, there is an accumulation of material depending on the cycle phase and the wavy bead course. In comparison, the wall structure welded with the CMT-ADV mode shows a uniform height profile.

Regarding the final contour, the multi-layer wall produced with the CMT-PADV arc process has the most regular contour in the cross-section. The measured surface irregularity of this wall is the lowest at 0.89 mm and the material utilization is the highest at 80%. On the other hand, the surface irregularity of the CMT-ADV was the highest at 1.4 mm and the material utilization was the lowest at 73%. This is mainly due to the displacement, which is clearly visible in the cross-section.

### 4.4. Characterization of the Temperature–Time-Regimes of Multi-Layered Structures

Wire- and arc-based additive manufacturing results in complex temperature–time profiles within the manufactured object. When a new layer is deposited on top, the previously placed layer is partially melted and undergoes repeated temperature changes. The resulting temperature–time profile during the additive manufacturing depends, among other things, on the thermal conductivity of the base material and filler material, the heat input, the dwell time between the deposition of individual layers or the interlayer temperature and the size or volume of the part.

**Figure 12:**
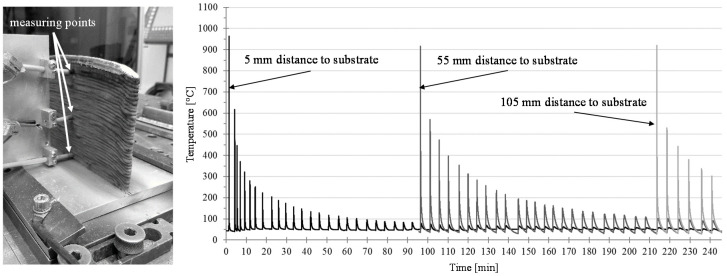
(**Left**) Representation of the temperature measuring points and (**Right**) the recorded temperature–time curve for the different temperature measuring points.

The location-related development of the microstructure, and consequently, the location-dependent mechanical properties of an additive-manufactured component are directly related to the temperature–time curve. The temperature–time curve recorded during the generation of the structure using the CMT-ADV arc mode is shown in Figure 12b. On the left side (Figure 12a), the arrangement of the measuring points in the experiment is illustrated. A temperature peak indicates the time of a new layer generation. It is observed that with an increasing number of layers, the maximum temperature at the surface of the layer from which the temperature was recorded by a thermocouple decreases. It is also indicated that, regardless of the buildup height, the temperature of the measured layer surface was heated over 300 °C by the following five welded layers. Depending on the structure height, a temperature of 100 °C is no longer surpassed after 20 to 24 subsequent layers. Accordingly, an influence on the microstructure may be expected, since the increased temperature causes grain growth.

With an increasing construction height or number of layers, the welding pause time, indicated by the time intervals between the two temperature peaks, increases. Using the recorded temperature–time curves, cooling rates *C*_500/300_ (K/s) for cooling from 500 to 300 °C were determined for the layers under investigation (see Equation (5)). Herein, the temperature difference Δ*T* between 500 °C and 300 °C was divided by the time Δ*t*, which is required for the cool down:

(5) $$C_{500/300}=\frac{\Delta T}{\Delta t}\left[\frac{K}{s}\right]=\frac{T_{500\;{}^{{}^{\circ}}C}-T_{300\;{}^{{}^{\circ}}C}}{t_{500\;{}^{{}^{\circ}}C}-t_{300\;{}^{{}^{\circ}}C}}\left[\frac{K}{s}\right]$$

**Figure 13:**
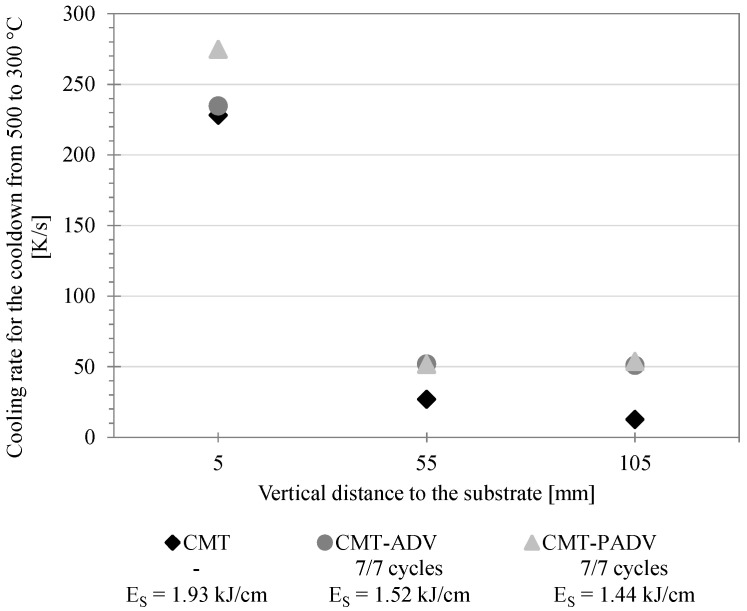
Representation of the cooling rate for cooling from 500 to 300 °C as a function of the vertical distance of the measuring point to the substrate plate and of the arc mode.

With the aid of these, differences in cooling behavior were analyzed as a function of the position of the measuring point in the walls, the energy input per unit length and the arc mode. The results are shown in Figure 13. It is obvious that the cooling rate in the immediate area of the substrate was about four to five times as high as at the other measuring points. The high cooling rate at the beginning is due to the high temperature gradient and the high heat conduction to the substrate. While during the application of the first layers, with a small vertical distance to the substrate, the heat transfer occurs mainly in the form of heat conduction in the direction of the substrate, and the heat transport occurs increasingly by convection and thermal radiation as the wall height increases. However, these two heat transfer mechanisms have a much smaller effect than heat conduction. With an increasing wall height, the heat conduction decreases and therefore heat accumulation along the direction of the substrate occurs. Decreasing heat conduction and heat accumulation leads to a reduction of the cooling rate [31]. However, this effect also decreases as the number of layers increases. For this reason, the cooling rates for the two measuring points at 55 and 105 mm are almost identical. At the same time, there is a slight dependence on the applied arc mode and the correspondingly different energy input per unit length.

With regard to the required time to build up the structures, significant differences could be observed (see Table 2). With a time span of 5 h and 20 min, the most time was consumed during the CMT process. By using the CMT-ADV arc mode, this time was reduced by approx. 22% to 4 h and 10 min due to the lower heat input. The shortest set-up time was achieved with the CMT-PADV arc mode, which also has the lowest energy input per unit length and thus the lowest heat input. Compared to the CMT mode, the duration was reduced by approx. 33% to 3 h and 35 min.

### 4.5. Analysis of the Porosity of Multi-Layered Structures

**Figure 14:**
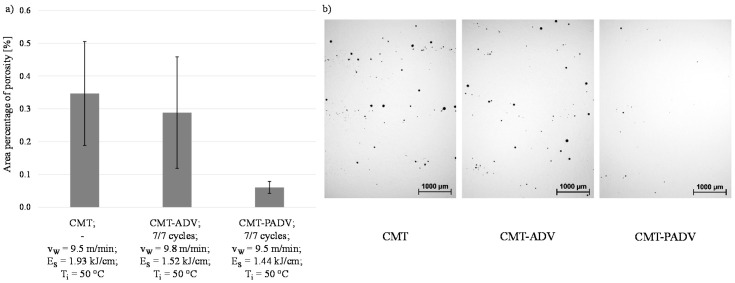
(**a**) Area percentage of porosity in the multilayer walls as a function of the arc mode and (**b**) the samples taken from the central area of the walls for the porosity analysis.

As already observed in the preliminary tests, it has been confirmed in the WAAM of multilayer wall structures with AlMg5Mn, that a reduction of the energy and thus the heat input leads to a decrease in porosity (see Figure 14a). The differences in the cross-sections of the central area of the wall structures are shown in Figure 14b, illustrated by the microscopic images for the analysis of porosity.

The observed porosity was the highest in the standard CMT process, as in the five-layer walls, in comparison with the other two processes. Moreover, 0.347% porosity as a proportion of the total cross-sectional area was found, which was more than twice as high as the porosity observed for the same welding parameters in the five-layer wall. When using the CMT-ADV arc mode, the area percentage of porosity of 0.288% was even more than four times as high as with the same parameters in the five-layer wall. While the porosity in the first five to six layers of the wall was unchanged compared to the area fraction of five-layer walls, the observed porosity in the central and upper area of both walls was significantly higher. As a result of the inhomogeneous pore distribution, the scattering of the measured values of both samples was comparatively large. In contrast, the porosity in the sample welded with CMT-PADV was on average 0.06%, even slightly lower than in the five-layer wall. The pores were also homogeneously distributed along the height of the wall. In all three structures, the pores occurred more often at the layer boundaries.

### 4.6. Microstructural Analysis of Multi-Layered Structures

**Figure 15:**
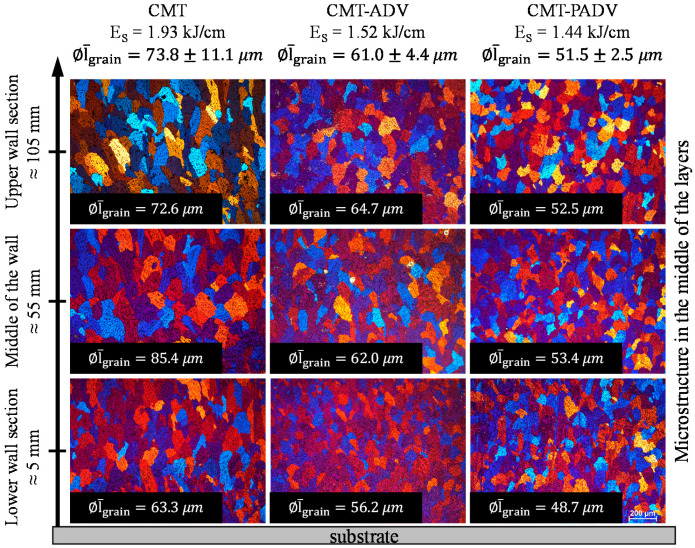
Comparison of the grain size in the center of the layer depending on the vertical distance to the substrate when using the CMT, CMT-ADV and CMT-PADV mode and the indication of the average linear grain size in the respective range.

The observed microstructure and grain size varied with the used arc mode. To characterize the grain size, the average linear grain size was measured in the lower wall section (between the second and third layer), in the center of the wall and in the area of the third and fourth last layer, respectively, in the center of the layer and at the transition between the two layers. Figure 15 compares the microstructure observed in the center of the layers for the three arc modes in dependence of the vertical distance to the substrate with a detail of the average linear grain size *l_grain_* for the different layers and the whole structure.

The line energy of 1.93 kJ/cm and consequently the heat input was the highest in the CMT arc mode compared to the CMT-ADV and CMT-PADV methods. In addition, the standard CMT process does not involve any polarity reversal, so most of the heat is conducted into the base material. As a result, when applying new layers, the penetration into the previously applied layer or the amount of re-melting was significantly increased compared to the other arc modes. Furthermore, since the tests were carried out without preheating, the temperature gradient was larger for the first layers. Due to the high thermal conductivity of aluminum, the interlayer temperature could be kept constant at 50 °C after a few layers. Since grain growth is primarily aligned along the largest temperature gradients, a microstructure with predominantly columnar grains, which have grown perpendicular to the substrate, appears in the lower wall section. The average grain size measured in the CMT process was 63.3 µm in the lower wall section.

Once the interlayer temperature was reached, the temperature gradient was highest in the CMT process compared to the other two processes due to the high heat input. As a result, columnar grain growth also took place in the center and upper wall sections along the melt lines. As a result of the preheating that resulted from the previously applied layers, the temperature gradient along the fusion lines decreased. As a result, the observed columnar grain structure was significantly coarser at the layer boundaries.

While the heat is quickly and effectively dissipated through the previously applied layers in the direction of the substrate during the application of the first layers, the heat flow to the substrate slows down with the increasing wall height due to the increase in distance to the heat source. Due to the reduced heat conduction, the cooling rate decreased (see Section 4.3). As a result of a smaller temperature gradient and a higher crystallization speed, the grains show an undirected globulitic growth in the center of a layer and due to the higher cooling rate in the center and upper wall section. However, the area was comparatively narrow since a large percentage of the layers was remelted when new layers were applied. Only the uppermost layer showed a distinct equiaxial dendritic microstructure. The decrease in the cooling rate resulted in a significantly coarser microstructure in the center and upper wall area (see Figure 15). The measured grain size was 85.4 µm in the middle wall section and 72.6 µm in the upper part of the wall.

The multilayer samples welded using the CMT-ADV and CMT-PADV arc modes with alternating polarity showed a significantly finer microstructure with a cellular grain structure compared to the welded sample using the standard CMT method. The reason for this observation is, on the one hand, the periodic change of the electromagnetic force and its direction by the polarity changes. This results in a flow in the melt pool in the form of a strongly oscillating turbulence of the melt. The dendrites growing directionally along the melt line break apart and the broken dendrite arms act as heterogeneous nucleation sites. A finer grained microstructure results from a melt solidifying uniformly at several points [15]. On the other hand, a larger part of the existing Al_3_Ti phase particles remains because of the lower heat input and the high melting point. These particles result from the titanium admixtures in the welding wire of up to 0.08%. They act as heterogeneous nucleation particles during solidification and lead to a refinement of the microstructure [9].

The measured grain size using the CMT-ADV method was 56.2 µm near the substrate. In the center and upper wall area, the grain size was 62.0 and 68.7 µm, respectively, due to a lower cooling rate. In the CMT-PADV process, the finest globulitic grain structure was observed in comparison to the other two processes, with an average grain size of 48.7 µm in the lower wall section, 53.4 µm in the middle wall section and 52.5 µm in the upper wall section. This can be explained by the fact that the grain size and morphology correlate with the heat input. A lower heat input results in a finer grain structure and in comparison, the CMT-PADV process has the lowest energy input. At the same time, the pulsed current in the CMT-PADV mode reinforces the effect of turbulence in the melt pool, since the electromagnetic force is directly proportional to the current intensity.

### 4.7. Analysis of Mechanical Properties of Multi-Layered Structures

**Figure 16:**
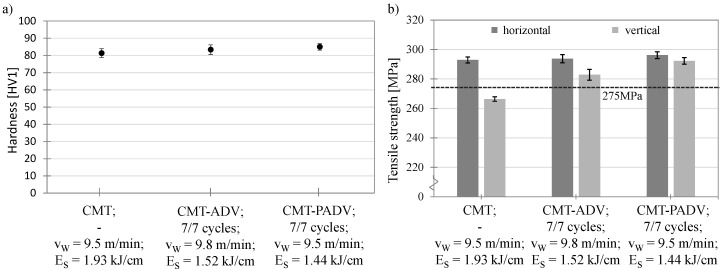
(**a**) Representation of the average hardness measured along the structure in the buildup direction as a function of the arc mode and (**b**) the representation of the tensile strength of the samples taken vertically and horizontally as a function of the arc mode and the manufacturer’s specification of the tensile strength of the pure weld metal (dashed line).

Regarding the mechanical properties of the welded structures, a hardness measurement was carried out along the height of the structure. The average hardness values are plotted in Figure 16a as a function of the applied arc mode. The structure produced with the CMT process has the lowest hardness value of 81.4 HV1. The produced structures using the CMT-ADV or CMT-PADV arc mode have slightly higher hardness values of 83.4 or 85 HV1. A possible explanation is the lower energy and heat input and the resulting finer microstructure for the two arc modes, because the amount of grain boundaries and hence the resistance against penetration is higher. In the CMT-ADV and CMT-PADV process, the hardness curve over the structure height decreases slightly with increasing distance to the substrate up to a structure height of 95 mm and increases slightly in the further course to the end of the structure. This gradient agrees with the analysis of the microstructure (see Section 4.5), since a finer-grained structure is present in the lower area than in the areas above. An increase in hardness in the uppermost layers can be attributed to the fact that less heat was introduced into these layers by fewer subsequent weld beads.

**Figure 17:**
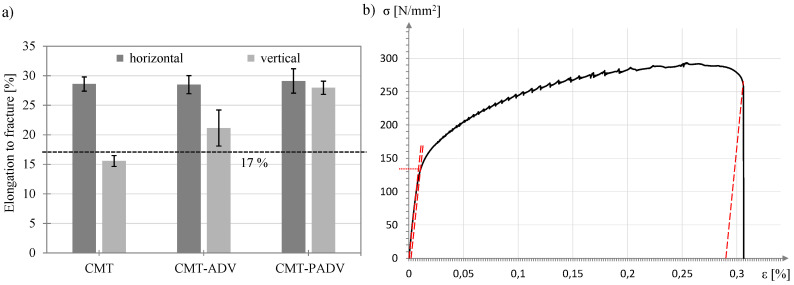
(**a**) Representation of the elongation to fracture of the samples taken vertically and horizontally as a function of the arc mode and the manufacturer’s specification of the elongation to fracture of the pure weld metal (dashed line) and (**b**) the stress–strain diagram for a horizontally taken tensile specimen in the CMT-ADV manufactured structure with dynamic strain ageing.

Figure 16b shows the tensile strength of the sample structures produced with the different arc modes. In each case, the samples taken in the horizontal direction (welding direction) and vertical direction (buildup direction) are shown. The dashed line at 275 MPa represents the manufacturer’s specification of the tensile strength of the pure weld metal. The highest tensile strength in the horizontal direction of 296.1 ± 2.3 MPa was determined in the CMT-PADV process and differs only slightly from the values of the CMT-ADV (293.8 ± 2.7 MPa) and CMT (292.8 ± 2.0 MPa) arc modes. As the results are within the scattering range of each other, the tensile strength values in the horizontal direction can be considered equivalent. Furthermore, the small standard deviations in all three methods allow conclusions to be drawn about homogeneous strength properties in the horizontal direction. This is reflected in the determined elongations to fracture. For the horizontally taken test specimens, elongations to fracture of 28.6% ± 1.2% for the CMT, 28.5% ± 1.5% for the CMT-PADV and 29.1% ± 2.1% for the CMT-PADV modes were achieved (see Figure 17). No differences in ductility between the arc modes can be observed. The values exceed the reference value for the pure weld metal of 17 % elongation at fracture given by the manufacturer, represented by the dashed line in Figure 17a.

In contrast, the tensile strength and elongation to fracture of the samples taken from the vertical direction are significantly lower for the CMT and CMT-ADV processes than for the horizontally arranged samples. The lowest tensile strength of 266.4 ± 1.5 MPa and elongation to fracture of 15.6 ± 0.9% could be determined for the CMT process. For the CMT-ADV arc mode, these characteristic values are 282.2 ± 5.3 MPa, and 21.2% ± 3%, respectively. Furthermore, there is no decrease in stress in the stress–strain diagram shortly before specimen failure. Thus, the deformation capacity in the vertical direction of the specimens manufactured with the CMT and CMT-ADV processes is reduced. Accordingly, the anisotropic strength and ductility properties are present in the structures, and the direction of loading has an influence on the maximum bearable force or stress. Only with the CMT-PADV process an almost isotropic behavior in the structure could be determined. The measured tensile strength in the vertical direction is 292.3 ± 2.2 MPa and thus differs only by 2.2 MPa from the tensile strength in the horizontal direction. The same applies to the values determined for elongation to fracture. At 28 %, the elongation to fracture is approx. 1% lower in the vertical direction than in the horizontal direction. In addition, in the samples of the CMT-PADV mode, no significant differences in the failure pattern or the stress–strain curve could be found.

Since a concentration of porosity was found at the layer boundaries, the anisotropy between the vertical and horizontal directions can be attributed to the cross-sectional weakening by the pores [10] and crack initiation by stress concentration at the pores [32]. In addition to reducing the tensile strength, this also has negative effects on ductility. As a result, the tensile strength and elongation to fracture in the vertical direction correlate with the determined porosities, which were highest in the CMT process, followed by the CMT-ADV process. With the CMT-PADV arc mode, the lowest porosity and the finest microstructure could be determined due to the lowest heat input. Accordingly, an almost isotropic strength and ductility properties were measured.

Figure 17b shows the stress–strain diagram using the example of a horizontally taken tensile specimen of the wall structure produced by CMT-ADV. From the diagram, the 0.2% proof stress at approx. 120 MPa can be determined and a breaking elongation of approx. 29% results. Furthermore, a zigzag stress curve is observed, which occurred in the stress–strain diagram of every sample.

This phenomenon is due to the Portevin-Le-Chatellier-(PLC) effect. The occurrence of the effect is a typical characteristic of AlMg(Mn) alloys and depends, among other factors, on the magnesium content. At a magnesium content above 0.5%, the dynamic strain ageing increases. The dissolved magnesium atoms remain in the vicinity of the dislocations and block their movement. This leads to an increase in the yield stress. This rises to a critical value, above which the dislocations break away from their blockade of dissolved magnesium atoms. This results in a sudden drop in the yield stress. After tearing, the mobile dislocations again collide with other blocked dislocation walls and the magnesium atoms again impede movement, which leads to the blocking of the movable dislocations. The resultant stress deflection increases as the dislocation density increases with deformation [27].

As shown in Table 3, the determined mechanical properties of the structure generated by the CMT-PADV process exceed the minimum values specified by the manufacturers for the pure weld metal in terms of tensile strength, 0.2% proof stress and elongation to fracture.

## 5. Conclusions

In this study, the arc-based additive manufacturing of the aluminum alloy AlMg5Mn was investigated. The controlled and energy-reduced short arc technology in form of the CMT process and its variations, CMT-advanced and CMT-pulse advanced, were applied for this purpose.

In the preliminary tests, the different arc modes CMT, CMT-ADV and CMT-PADV were examined and their influence on the energy input, the near-net shape and the porosity were analyzed by varying the welding speed. For this reason, small volume wall structures were generated. Furthermore, the influence of the number and the ratio of different pole changing cycles in the CMT-ADV process was part of the investigations. Different strategies to avoid the bonding defects and to improve the final contour proximity while reducing porosity were investigated. For this purpose, the welding speed, the wire feed and the shielding gas composition were varied. Compared to the reference structure, only one buildup strategy was able to achieve a simultaneous increase in the final contour proximity and the reduction of porosity. For this purpose, the welding speed was reduced by 50% in the first layer and a mixture of 70% argon and 30% helium instead of pure argon was used within the first two layers.

Subsequently, in the main trials, three large volume wall structures with a total height of 110 mm were built up layer by layer using the arc modes CMT, CMT-ADV and CMT-PADV, and then analyzed regarding geometric properties, porosity, microstructure and mechanical properties tensile strength and hardness. The results are summarized as follows:

- With CMT-PADV, structures with the least surface irregularity and the highest material utilization in cross-section could be manufactured.
- With the CMT-ADV process, the most homogeneous wall structure height could be produced, i.e., the wall drop at the beginning or end of the structure is the lowest.
- The differences in energy input result in shorter cooling times for the CMT-ADV and the CMT-PADV arc mode during the buildup process. As a result, the buildup time could be reduced by approx. 22% for the CMT-ADV process and by 33% for the CMT-PADV process compared to the standard CMT process. In addition to the technical or material advantages, this results in a further economic advantage when using the CMT process variations by reducing the buildup time.
- A strong correlation between the microstructure and the energy input with regard to both the grain size and porosity was shown. The CMT-PADV process achieved the lowest energy per unit length of 1.44 kJ/cm and thus had the finest grain structure. The porosity was reduced to 0.06%.
- Regarding the hardness in the direction of the buildup, a slight influence of the applied arc mode could be determined. This was in the range of 81.4 HV1 for the CMT and 85 HV1 for the CMT-PADV. Accordingly, the hardness increased slightly with the reduced energy input.
- About the tensile strength tests, it was shown that the characteristic values fluctuated in the horizontal direction by the value of 294 ± 2 MPa, independent of the arc mode. Due to the porosity concentrating at the layer boundaries, anisotropic properties between the horizontal and the vertical loading direction were observed in the CMT and the CMT-ADV processes. The tensile strength was 26.4 and 19.8 MPa lower in the vertical direction, respectively. In the CMT-PADV mode, an almost isotropic behavior could be concluded with tensile strengths of 294.5 MPa in the horizontal direction and 292.3 MPa in the vertical direction. This was attributed to the lowest porosity and the finest grain structure.

## Figures and Tables

**Table 1:** Chemical composition of the substrate and the welding wire (wt %).

Material (Function)	Chemical Composition in wt %								
	Si	Fe	Cu	Mn	Mg	Cr	Zn	Ti	Al
EN AW-5754A H111 (substrate)	0.4	0.4	0.1	0.5	2.6–3.6	0.3	0.2	0.15	bal.
S Al 5556 (welding wire)	0.06	0.18	0.009	0.7	5.3	0.08	0.01	0.08	bal.

**Table 2:** Comparison of the buildup time of the different arc modes for the large-volume wall structures.

Welding Process	Buildup Time (min)	Time Savings (%)
CMT	320	–
CMT-ADV	250	−22
CMT-PADV	215	−33

**Table 3:** Mechanical properties of the weld material and the tested specimen (CMT-PADV).

Mechanical Properties	Standard Values for the Weld Material [33,34]	Wire Arc Additive Manufacturing (WAAM) Using CMT-PADV
Rm (MPa)	275	294.2 ± 3
Rp0.2 (MPa)	125	131.8 ± 4.4
A (%)	17	28.5 ± 1.5
